# Response dynamics of phosphorelays suggest their potential utility in cell signalling

**DOI:** 10.1098/rsif.2010.0336

**Published:** 2010-08-11

**Authors:** Attila Csikász-Nagy, Luca Cardelli, Orkun S. Soyer

**Affiliations:** 1 Microsoft Research—University of Trento Centre for Computational and Systems Biology (CoSBi), Piazza Manci 17, Povo (Trento) 38100, Italy; 2 Microsoft Research Cambridge, 7 J J Thomson Avenue, Cambridge CB3 0FB, UK; 3 Systems Biology Program, College of Engineering, Mathematics and Physical Sciences, University of Exeter, Exeter EX4 4QF, UK

**Keywords:** two-component signalling, ultrasensitivity, computational modelling, cross-talk, noise

## Abstract

Phosphorelays are extended two-component signalling systems found in diverse bacteria, lower eukaryotes and plants. Only few of these systems are characterized, and we still lack a full understanding of their signalling abilities. Here, we aim to achieve a global understanding of phosphorelay signalling and its dynamical properties. We develop a generic model, allowing us to systematically analyse response dynamics under different assumptions. Using this model, we find that the steady-state concentration of phosphorylated protein at the final layer of a phosphorelay is a linearly increasing, but eventually saturating function of the input. In contrast, the intermediate layers can display ultrasensitivity. We find that such ultrasensitivity is a direct result of the phosphorelay biochemistry; shuttling of a single phosphate group from the first to the last layer. The response dynamics of the phosphorelay results in tolerance of cross-talk, especially when it occurs as cross-deactivation. Further, it leads to a high signal-to-noise ratio for the final layer. We find that a relay length of four, which is most commonly observed, acts as a saturating point for these dynamic properties. These findings suggest that phosphorelays could act as a mechanism to reduce noise and effects of cross-talk on the final layer of the relay and enforce its input–response relation to be linear. In addition, our analysis suggests that middle layers of phosphorelays could embed thresholds. We discuss the consequence of these findings in relation to why cells might use phosphorelays along with enzymatic kinase cascades.

## 1. Introduction

**Figure 1.**
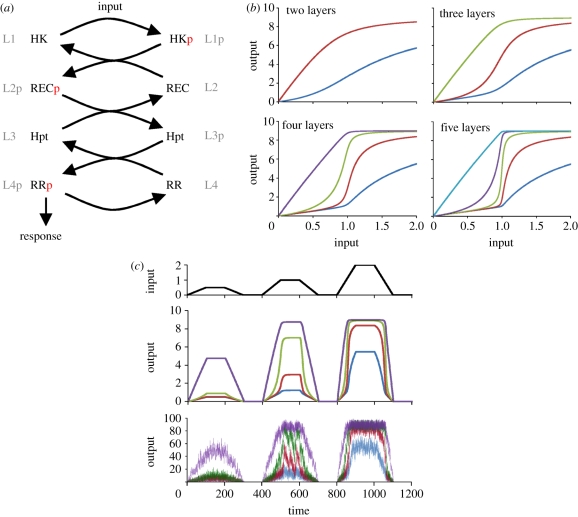
Dynamics of linear phosphorelays. (*a*) Cartoon representation of the linear phosphorelay considered. The phosphotransfer reactions among each layer are shown for a four-layer relay. Note that other configurations of specific proteins (RR, HK, etc.) are possible that would lead to the same dynamical effects. The generic relay structure is indicated by referring to different layers as L1, L2, etc. (*b*) Steady-state input–response curves are shown for each layer in relays with increasing relay length (number of relays indicated on top of each panel). Dark blue lines, L1p; brown lines, L2p; green lines, L3p; purple lines, L4p; light blue lines, L5p. (*c*) Time course showing system response (phosphorylated form of each layer), obtained from deterministic (middle) and stochastic (bottom) models of a four-layer system. Changes in input during the time course of simulation are shown in the upper panel. For the stochastic model, the input values are multiplied by 100 (§4).

Organisms employ a variety of signalling systems to sense and react to their environment. One of these, commonly found in bacteria, lower eukaryotes and plants, is the so-called phosphorelays [1,2]. Phosphorelays are extended two-component systems [3] where there are several intermediate proteins between a histidine kinase (HK) and a response regulator (RR), resulting in a structurally linear cascade (figure 1*a*). Histidine kinases are signalling proteins with autophosphorylation ability, which is usually controlled directly or indirectly by external signalling molecules. Once autophosphorylated, an HK can transfer this phosphate group to an intermediate protein in a phosphorelay. These intermediate proteins are usually His-containing phosphotransfer proteins (Hpt) or proteins with receiver domains (REC) similar to those found on RRs [3]. In some systems, such as that controlling virulence in *Bordetella* [4], the relay function is achieved with a single protein that contains these multiple domains. Phosphorelays terminate with an RR, which in its phosphorylated form is involved in the generation of a physiological response. Such responses mediated by phosphorelays include the control of sporulation [5], virulence [4], stress responses [6] and cytokinin signalling [7], appearing in both prokaryotes and eukaryotes. Interestingly, all these characterized systems have a similar structure composed of four layers usually arranged as HK–REC–Hpt–RR, despite differences in biophysical implementation [8]. In each case, the intermediate proteins (or protein domains) shuttle a single phosphate group all the way from an initial HK down to a final RR.

Despite their widespread use, we are still far from a full understanding of the signal-processing capabilities of phosphorelays. What dynamical advantages, if any, do phosphorelays offer? Why are there structural similarities among different systems, especially with regard to relay length? Why are phosphorelays employed in processing certain signals, while other signals are processed by a single HK–RR pair? Similarly, why do certain eukaryotes use these systems in conjunction with enzymatic kinase cascades? While intermediate proteins in a phosphorelay can provide additional points for signal integration [5], there are other systems where phosphorelays clearly lack any known signal integration function [4]. It is not clear how these differences translate to functional roles or dynamical properties of these systems. Towards a better ability to answer these questions, our aim here is to achieve a general understanding of structure and dynamics in phosphorelay signalling. To this end, we develop a generic model of a phosphorelay and characterize its steady-state response dynamics at each layer. We repeat this analysis for relays of different length and under different assumptions regarding the functionality of the kinase at the top of the relay (i.e. a bifunctional kinase, see below). Further, we run deterministic and stochastic simulations to analyse how relay length alters the effects of cross-talk and noise in these signalling systems.

## 2. Results

Phosphorelays starting with an HK and ending with an RR constitute one of the signalling systems found in microbes and plants. Here, we analyse the properties of these diverse systems from a response dynamics perspective. As the number of larger systems that can be constructed using the modular structure of two-component systems is immense, we concentrate here only on linear relays where no transcriptional (or other) feedbacks occur (figure 1*a*). We construct generic mathematical models for relays of varying lengths (§4). Using these models, we first analyse the response dynamics of these systems. In particular, we derive steady-state response levels (i.e. phosphorylated protein concentrations) at each input level, resulting in so-called input–response curves (also known as signal–response or dose–response curves). Interestingly, we find that increasing relay length leads to increasingly more ultrasensitive responses at intermediate layers (figure 1*b*). As one would expect from ultrasensitive responses, the intermediate layers show a switch-like response, generating low (high) responses for inputs below (above) a threshold (figure 1*c*, top two panels). The response coefficient of the most ultrasensitive layer (which measures the ratio between input levels resulting in 10% and 90% activation at this layer) saturates with relay length and already gets close to the minimum possible value of one (maximal ultrasensitivity) at a relay length of four (electronic supplementary material, figure S1). Unlike the intermediate layers, the phosphorylated protein concentration at the final layer is increasing with increasing input before it saturates. With increasing relay length, the input–response curve for this layer becomes linear, while its transition from linear to saturated sharpens (figure 1*b*). The latter effect results in increasing sensitivity, calculated as *Δ*L4p/*Δ*input summed over the signal (i.e. input) range from 0 to 2. Both effects seem to saturate around four to five layers (electronic supplementary material, figure S2).

The ultrasensitivity at intermediate layers is a surprising feature of the phosphorelay and is in contrast to eukaryotic kinase cascades, where any ultrasensitivity that can be achieved is shown to increase towards the end of the cascade and is maintained at the final layer [9,10]. We find that the reason for these differences lies with the fact that phosphorelays shuttle a single phosphate group among different proteins rather than using separate ATP for each layer, as enzymatic kinase cascades do. At low input levels, the phosphate group from the HK at the top of the relay is shuttled all the way down to the last layer. In other words, at these input levels, the relay simply acts as a phosphate channel; all the intermediate layers are pushed back to their unphosphorylated state simply by transferring the phosphate group to lower layers. Once the input level reaches a point where the last layer is saturated, the layer above it starts to accumulate in its phosphorylated state. This dynamics cascades backwards through the relay with proteins at layers closer to the top of the pathway being the last to accumulate in their phosphorylated form. The result is nonlinear input–response curves (i.e. ultrasensitivity) for phosphorylated forms of the proteins at intermediate layers (figure 1*b*). In other words, for each protein in the relay, those below it act as a threshold generator by removing its activating phosphate group. Performing an extensive robustness analysis (as in Barkai & Leibler [11]), we find that this effect is achieved under a large range of parameters (electronic supplementary material, figures S3 and S4). In particular, the second layer of a four-layer system maintains ultrasensitivity under a wide range of kinetic rates and protein concentrations, suggesting that relay structure rather than kinetic parameters is the key factor in the emergence of ultrasensitivity in the system. Lowering the total protein concentration in the first layer has a strong negative effect on ultrasensitivity (electronic supplementary material, figure S3B), but this effect is due to resulting slow input, and could be compensated for (i.e. ultrasensitivity can be regained) by decreasing self-dephosphorylation of the last layer.

**Figure 2.**
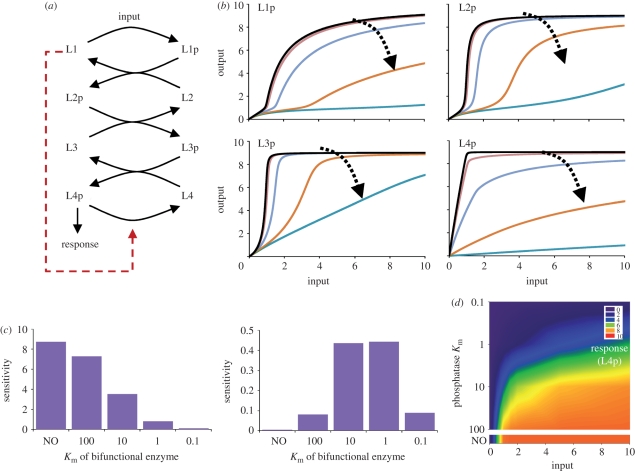
Effects of a bifunctional HK. (*a*) Cartoon representation of a four-layer relay, with a bifunctional HK sitting at the top and capable of dephosphorylating L4p (§4 and electronic supplementary material, equation (S1)). (*b*) Effect of the Michaelis constants (*K*_m_) of the bifunctional HK on input–response curves. Dashed arrows show the trend how the responses in each layer (noted on each panel) are changing with increasing dephosphorylation efficiency of the bifunctional HK. *K*_m_=0.1 (dark blue lines), 1 (orange lines), 10 (light blue lines), 100 (brown lines), NO (black lines). (*c*) Sensitivity in L4p as *K*_m_ of the bifunctional enzyme is varied. Sensitivities were calculated between input levels 0–2 (left panel) and 2–10 (right panel) from the changes in the response and input (i.e. *Δ*L4p/*Δ*input) in the investigated input regimes. In other words, sensitivity corresponds to the slope of the input–response curve for L4p (as shown in (*b*)). (*d*) Heat map of input–response relation of a four-layer relay, with varying values of the Michaelis constant (*K*_m_) of the bifunctional HK in layer 1 (§4 and electronic supplementary material, equation (S1)). Bottom plot with the NO label shows the heat map for a system without the phosphatase activity of the HK.

In line with the mechanism of how ultrasensitivity is generated in the relay, we find that the threshold and sharpness of the input–response curves at intermediate layers can be tuned by the dephosphorylation rate of the last layer (electronic supplementary material, figure S5). Dephosphorylation of the last layer can occur through hydrolysis activity intrinsic to RRs and through dephosphorylation by a specific phosphatase or a bifunctional HK. In particular, bifunctional HKs are common in single HK–RR pairs, where they can both phosphorylate and dephosphorylate the RR [12]. There is one reported case of bifunctional HKs in phosphorelays, where the HK at the top of the relay transfers its phosphate group to the next layer but dephosphorylates the RR at the bottom of the relay [7]. Based on that study, we model the effect of a bifunctional HK at the top of the relay as an enzymatic dephosphorylation of the final layer (L4p) by the first layer (L1) (§4 and figure 2). We find that increasing the efficiency of this enzymatic reaction flattens input–response curves of all layers and pushes the threshold point to higher input values (figure 2). This in turn reduces sensitivity of the final layer in the same input regime (from 0 to 2), but extends the range of inputs where the system can respond. Slowing down dephosphorylation of the last layer (either by making L1 less efficient or by reducing the self-dephosphorylation rate of L4p) has the exact opposite effects and reduces the effective signalling regime but makes the system more sensitive in this reduced regime (figure 2 and electronic supplementary material, figure S5). Bifunctional HKs are also shown to be important for ensuring robustness in the output of a signalling system, against variations in protein concentrations [13,14]. In particular, inclusion of a bifunctional enzyme in a phosphorelay can allow the system to satisfy certain structural features that ensure ‘absolute concentration robustness’, that is robustness of the output to variations in the concentrations of system parts [15]. In line with this theory, we find that the inclusion of a bifunctional HK in the presented model allows absolute concentration robustness if the self-dephosphorylation rate of the last layer is small compared with dephosphorylation by the bifunctional enzyme (electronic supplementary material, figure S6).

**Figure 3.**
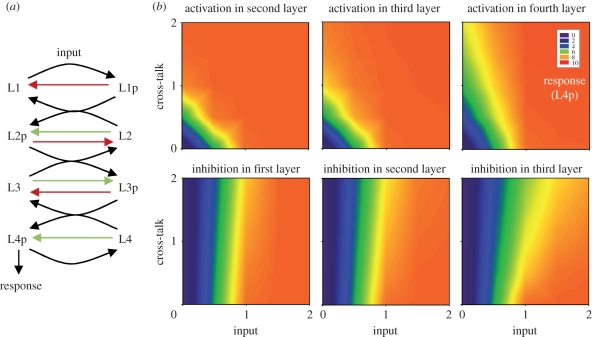
Possible cross-talks in a phosphorelay. (*a*) Four-layer phosphorelay with cross-activation—‘talk in’ (green)—and cross-inhibition—‘talk out’ (red). Note that these cross-talk reactions are modelled as self-phosphorylation (*k*_23_, *k*_33_, *k*_43_) and self-dephosphorylation (*k*_22_, *k*_32_, *k*_42_) in the model (equation (4.1)). (*b*) Heat map showing response level given the input at the top of the relay and the secondary signal coming from cross-talk. The location where cross-talk occurs and its nature (i.e. activating or deactivating) are indicated on each panel (see also electronic supplementary material, figure S5).

One possible use for ultrasensitivity only at intermediate layers in the phosphorelay is to limit or exploit the effects of cross-talk. To explore this possibility, we considered all potential cross-talks in a four-layer linear phosphorelay (with a mono-functional HK). At each layer, cross-talk can occur as activation or inhibition, resulting in two cross-talk points for each layer. Cross-activation corresponds to cellular phosphate donors or proteins from other pathways phosphorylating any of the layers of the original phosphorelay (e.g. another relay ‘talks in’ to the investigated one via phosphorylation). Cross-inhibition corresponds to proteins from the original relay losing their phosphate to hydrolysis or transferring it to other pathways (i.e. investigated relay ‘talks out’ to another pathway). We modelled such cross-talk reactions as self-phosphorylation (reaction rates *k*_23_, *k*_33_, *k*_43_ in equation (4.1)) and self-dephosphorylation (reaction rates *k*_22_, *k*_32_, *k*_42_ in equation (4.1)) and quantified its effects by considering the system response in the presence of both the primary (i.e. coming from the HK at the top of the relay) and secondary (i.e. coming from cross-talk) inputs (figure 3*a*). We find that cross-inhibition is better tolerated in general compared with cross-activation (figure 3*b* and electronic supplementary material, figure S7). This is in line with the above-described general dynamics of the phosphorelay. Cross-activation adds on top of the primary input and causes the final layer to reach saturation at lower primary input levels (figure 3*b*, top row). This saturation effect is worsened as cross-talk happens closer to the final layer (electronic supplementary material, figure S7). Hence, for ‘talk-in’ to have any potential use as signal integration, it should operate closer to the final layer of the relay. In contrast to cross-activation, cross-inhibition reduces the rate of saturation at the final layer and allows the system to respond to the primary inputs in a wider range (figure 3*b*, bottom row). This effect is enhanced as inhibition occurs lower in the relay, but overall the effect of cross-inhibition remains milder compared with cross-activation (figure 3*b* and electronic supplementary material, figure S7). Hence, cross-inhibition might offer the possibility for signal branching (i.e. the phosphorelay producing secondary outputs, talking to other systems) without altering the response of the final layer. The best implementation for such a strategy would be to have phosphotransfer to other systems occur at higher layers of the relay. To further elucidate the effects of cross-talk on the steady-state response, we derived the sensitivity to the primary input under different points and strengths of cross-talk (electronic supplementary material, figure S8). We find that sensitivity is reduced both under cross-activation and cross-inhibition. In line with the above summary, this effect gets stronger as cross-activation (inhibition) occurs further up (down) the relay. These findings extend in general to systems with shorter relay length (electronic supplementary material, figure S8).

Ultrasensitivity at intermediate layers of a relay might also affect the noise properties of the system. Similar to noise in gene regulation [16], noise in signalling systems can result from the phosphorylation reactions inside the system (intrinsic noise), but also from amplification of any input noise (extrinsic noise). Previous studies have shown that such noise in a signalling system is directly proportional to its gain *g*, i.e. per cent change in response over per cent change in input [17]. In particular, intrinsic noise in a signalling protein *X* (i.e. fluctuations in its concentration) is expected to scale as 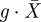, where 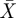 denotes the mean concentration level. It is not clear how these findings translate to phosphorelays. Moreover, it is not known how the relay structure affects the signal-to-noise ratio (SNR), a measure that could be thought of as the ability of a signalling system to convey meaningful information above background noise. Calculated as temporal mean concentration of a signalling entity (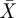) over noise (i.e. its standard deviation, *σ*), a low SNR would mean that such an entity fluctuates so widely that its mean value could not be taken as a reliable signal.

**Figure 4.**
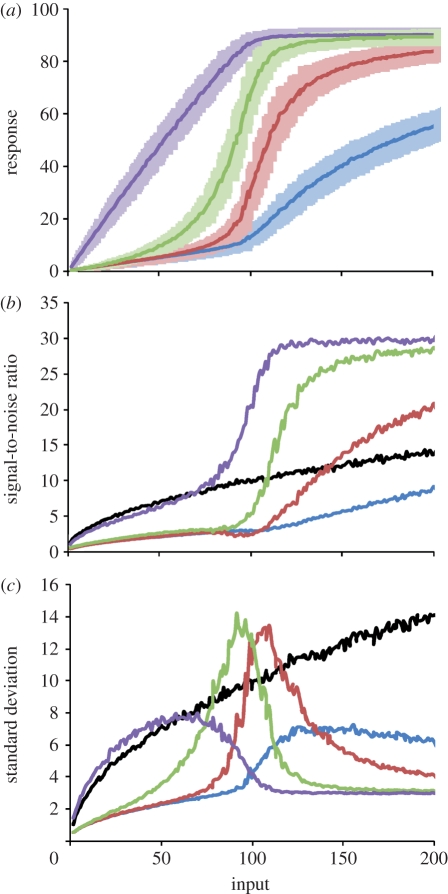
Noise tolerance of phosphorelays. (*a*) Average (dark colour curves) and standard deviation (lighter shading) of steady-state input–response curves for each layer in a four-layer system with noisy input. (*b*) SNR (§4) at each layer of a four-layer relay. The black line shows the SNR expected from a random Poisson process. See also electronic supplementary material, figure S10, which shows changes in the SNR from the last layer with changes in relay length. (*c*) Dependence of standard deviation on input strength at each layer of the four-layer system. All results were calculated from approximately 400 000 stochastic simulation data points at each of the 200 input levels. Blue lines, L1p; brown lines, L2p; green lines, L3p; purple lines, L4p, black lines, input.

To analyse noise properties in phosphorelays, we construct stochastic versions of the generic model presented above and run a large number of simulations at different input levels. This allows us to derive input–response curves as obtained previously from the deterministic models (§4 and figure 4*a*). Noting that this analysis shows that response dynamics in this system is well approximated by a deterministic model (compare figures 4*a* and 1*b*), we focus the analysis on noise properties. Using the data from figure 4*a*, we calculate noise and SNR for each layer and at different input levels. In line with earlier findings [17], we find that noise scales as 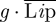, where 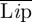 corresponds to the mean concentration of the phosphorylated protein from layer *i* (figure 4*c* and electronic supplementary material, figure S9). At low input levels, noise from the final layer tracks that of the input, while noise from intermediate layers is low. This is because at these input levels the relay acts as a phosphate channel as explained above, and there is no significant amounts of phosphorylated proteins in intermediate layers. As the input level increases, 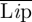 of intermediate layers starts to increase, leading to increase in noise. This increase peaks at the threshold, resulting in high fluctuations in intermediate layers for these input levels (figure 1*c*). Interestingly, the increase in noise at intermediate layers leads to a decrease in the noise from the final layer and results in a significant jump in SNR (figure 4*b*). We find that these improvements in the SNR of the final layer appears first within a three-layer system and seems to saturate at a relay length of four (electronic supplementary material, figure S10).

In summary, the noise in a relay is literally being pushed from layer to layer (figure 4*c*) as the input level increases. Just before the threshold point is reached, the noise in the final layer becomes lowest, while those of intermediate layers are highest. This noise peak at intermediate layers coincides with the input level where their response increases rapidly with small increases in input (i.e. ultrasensitivity). These noise dynamics translate into SNR for the final layer being always better than expected from a random Poisson process (black curve in figure 4*b*), while SNR of the intermediate layers improves only after the threshold input level is crossed.

## 3. Discussion

We undertook a systematic analysis of response dynamics in phosphorelays. Our key finding is that phosphorelays result in ultrasensitivity (i.e. input–response curves embedding thresholds) at intermediate layers of the relay. That ultrasensitivity could arise in two-component systems has been suggested before on grounds of specific mechanisms of dimerization and complex formation [18,19], but this study provides the first extensive description of this dynamics arising in linear phosphorelays. We note that this mechanism is significantly different from that described by Goldbeter and Koshland [20], where a target protein is activated and deactivated by a dedicated kinase and phosphatase, respectively, and in enzymatic fashion. In that mechanism, ultrasensitivity arises when both enzymes are highly efficient and act in the zero-order regime (i.e. are saturated). In contrast, the model presented here does not require enzymatic reactions. Rather, ultrasensitivity arises from the structure of the relay and from the fact that a single phosphate group is shuttled among different proteins.

We find that specific properties of the input–response curves in a phosphorelay can be tuned by the relay length and dephosphorylation rate of the last layer. At low input levels, the relay shuttles phosphate groups from the first to the final one, while above a certain threshold, saturation effects result in ultrasensitive responses at intermediate layers. This response dynamics reduces noise in the final layer over a wide range of input levels and increases noise at intermediate layers for input levels around the threshold point. Interestingly, a four-layer relay seems to be a saturating point for both increased sensitivity (at intermediate layers) and reduced noise (at the final layer), with longer relays not providing much improvement. In addition to these dynamic properties, we find that phosphorelays offer both the ability to tolerate and exploit cross-talk. In particular, we find that cross-inhibition at higher layers of the relay could allow signal branching without affecting response to primary inputs.

These findings are in agreement with several empirical observations from few characterized phosphorelays. In particular, they might provide an explanation for the observations that all relays described to date are four-layer systems [4,7,8] and that some relays contain phosphatases targeting at intermediate layers [8]. Furthermore, this analysis provides several interesting suggestions for how and why cells might be using phosphorelays. Firstly, the finding of ultrasensitive responses at intermediate layers suggests that these layers might act in their own right as a secondary output from the relay. Supporting this possibility, we find that in the phosphorelay controlling *Bacillus subtilis* sporulation, the second layer has the highest number of interaction partners (electronic supplementary material). Combined with the fact that ultrasensitive responses embed high noise, it is tempting to speculate that such secondary outputs, when combined with a feedback, could involve in stochastic switching at the population level and underlie bet-hedging strategies [21,22]. Secondly, the relay structure might have evolved to provide a more linear input–response relation at the final layer (figure 1*b*). While most biochemical processes introduce nonlinearities in input–response relation, it might be desirable that certain responses are linear in relation to the input. There is experimental indication, for example, that a linear increase in the final RR Spo0A is essential for proper response in the case of *B. subtilis* sporulation [23]. Thirdly, our findings on cross-inhibition suggest that phosphorelays might be highly suitable for allowing signal branching (i.e. phosphors being transferred to another relay) without detrimental effects to primary signalling. Alternatively, they might offer a mechanism for increasing signalling fidelity against unwanted cross-inhibition (e.g. loss of phosphors to hydrolysis). Finally, the existence of phosphorelays might relate to the fact that they use only one ATP molecule in contrast to most eukaryotic signalling cascades that consume high levels of ATP. Especially in adverse environmental situations that cause significant reduction in cellular ATP levels, phosphorelays might still be functional where enzymatic cascades might not be. This is an intriguing proposition given the observation that most phosphorelays found in microbes and plants to date are involved in stress-related responses [1]. The presented analysis shows that despite this key difference in ATP use, phosphorelays can still embed key dynamical properties as seen in eukaryotic systems, such as ultrasensitivity [20,24].

Experimental studies will be needed to further validate the findings and suggestions from this study. In particular, we envision that it could be possible to measure phosphorylation levels at intermediate layers of a phosphorelay to validate ultrasensitivity. Similarly, experiments can be devised to measure the effects of cross-inhibition. This would be an interesting avenue, as most works on cross-talk so far have concentrated only on cross-activation [12,25]. In addition to these specific directions, there are other potentially interesting avenues to be explored both theoretically and experimentally. For example, biochemical processes such as dimerization and regulation of total protein amounts at different layers of a phosphorelay might introduce additional response dynamics into the system as seen before [26–28]. Similarly, it would be interesting to analyse how feedbacks, as seen in some relays [29], could alter the global dynamical properties described here.

As with any other biological system, phosphorelays are intrinsically complex and the result of evolutionary processes. These two facts make it a daunting task to distil their key design properties that are significant for their functional role. Using generic mathematical models and theoretical analysis of response dynamics can play a useful and important part in this quest.

## 4. Methods

We construct generic models of phosphorelays by considering the core reactions of HK autophosphorylation and phosphate transfer. While modelling these reactions, we assume that protein complexes during phosphotransfer reactions are short-lived and are present only in negligible amounts. Hence, the core reactions we consider are autophosphorylation of an HK and subsequent phosphotransfer between HK, Hpt, REC and an RR.

We combine these core reactions to form relays of differing length; for a system with four layers (L1–L4), for example, we have L1 = HK, L2 = REC, L3 = Hpt and L4 = RR:
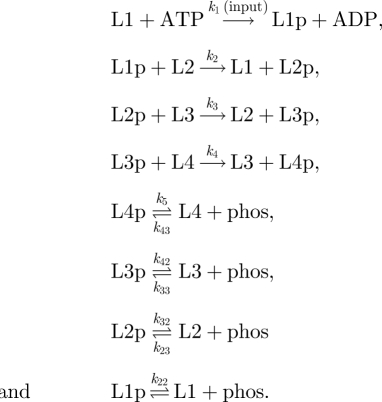



Besides the shuttling of the phosphate group (the top five reactions), these reactions also include cross-talk reactions represented as self-dephosphorylation and self-phosphorylation at each layer (except for the first layer where we consider only self-dephosphorylation). Note that for the main model, these reactions are considered to be negligible (i.e. the corresponding rates are set to zero). Based on these reactions, we write down ordinary differential equations and stochastic models describing system dynamics (electronic supplementary material). While writing these equations for the main model, we assume that the rate of autophosphorylation of HK is mediated by an external signal (input), ATP is not rate limiting, all reactions are unidirectional and the total level of proteins at each layer do not change during the time course of signalling. The final assumption would be satisfied if the phosphorylated and unphosphorylated forms of proteins at each layer have comparable degradation rates [30]. These assumptions allow us to write the ordinary differential equations describing a four-layer system as shown above as (using the L notation):
4.1
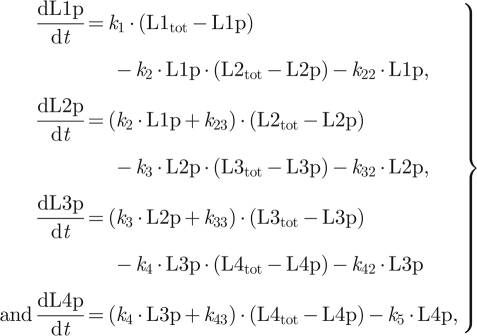

where L*i* and L*i*p denote the unphosphorylated and phosphorylated form of the protein at the *i*th layer and L*i*_tot_ denotes total protein concentration at that layer. For numerical simulations of equation (4.1), we set the total level of proteins at each layer to 10 and all reaction rates to 1.0 (arb. units). Implementation of equation (4.1) as an ODE model, executable by the XPPAUT (http://www.math.pitt.edu/~bard/xpp/xpp.html) or Oscill8 (http://sourceforge.net/projects/oscill8) software packages, is provided in the electronic supplementary material.

We analyse input–response dynamics of relays of varying length using the Oscill8 package. We numerically integrate the system to steady state at a fixed input (*k*_1_) level. We then numerically ‘follow’ this steady state, while changing the input, and trace the response (i.e. phosphorylated protein concentration) of the system. This analysis is equal to allowing the system to reach steady state under different input values. From the resulting input–response curves, we calculate the total system sensitivity and response coefficient. The former gives sensitivity (*Δ*L4p/*Δ*input) summed over the signal (i.e. input) range from 0 to 2 (figure 2), while the latter gives the ratio of input needed to reach 90 per cent of maximum response over the input needed to reach 10 per cent of response (i.e. the steepness of the response).

To test the effect of parameter choices on input–response curves and ultrasensitivity, we first derived an analytical steady-state solution of equation (4.1) by setting self-phosphorylation (*k*_23_, *k*_33_, *k*_43_) and self-dephosphorylation rates (*k*_22_, *k*_32_, *k*_42_) to zero (electronic supplementary material, equation (S2)). We then generated random parameter sets where total protein concentrations are drawn from a uniform distribution in the interval [0.01, 10] and kinetic rates are drawn from a uniform distribution in the interval [0.01, 2]. For each parameter set, we used the analytical solution to derive the input–response curve for L2p and calculated the response coefficient. In order to do so, we keep the total protein concentration of the second layer constant. This analysis shows that the system can achieve low response coefficients (i.e. high ultrasensitivity) for most kinetic rates and total protein concentrations (electronic supplementary material, figures S3 and S4).

Based on experimental evidence [7], we implement a bifunctional HK as one that, when in a non-phosphorylated form, de-phosphorylates the final layer (figure 2). We assume that dephosphorylation by a bifunctional HK occurs through complex formation and follows enzyme kinetics. The ordinary differential equations for the system with a bifunctional enzyme are shown in electronic supplementary material, equation (S1). To see the effect of a bifunctional HK with increasing enzymatic efficiency, we analyse system dynamics by varying the rate of HK–RRp complex formation (e.g. we set *K*_m_ to 0.1, 1, 10 and 100 by changing *k*_51_).

We implement cross-talk at various layers by increasing the corresponding self-phosphorylation (*k*_23_, *k*_33_, *k*_43_) and self-dephosphorylation rates (*k*_22_, *k*_32_, *k*_42_) (equation (4.1)).

To analyse noise in the system, we convert the above model to a stochastic reaction scheme, excluding the self-dephosphorylation and self-phosphorylation reactions and explicitly modelling HK activation by a signalling molecule (electronic supplementary material). We do so by using the SPiM modelling suite [31], which implements the Gillespie algorithm [32]. We set the number of proteins at each layer to 100 and correspondingly set the stochastic rates at 1.0 s^−1^ (for unimolecular reactions) and 0.1 s^−1^ (for bimolecular reactions; electronic supplementary material, equation (S5)). We set a lower rate for HK activation by the signal, to be able to more gradually increase the (discrete-level) input during stochastic simulation (note that this has no effect other than requiring a factor of 10 higher amount of input). To observe the equilibrium response of the phosphorelay, we progressively increase the amount of a signalling molecule from 0 to 200 (note that this corresponds to the 0–2 regime in the input in the deterministic model), reaching quasi-stability at each input level (each simulation produces approx. 80 000 000 data points). We then compute statistics of the corresponding data points, such as the SNR of the various layers. This analysis allows us to derive the behaviour of the system under just the irregular fluctuations intrinsic to any stochastic system (i.e. intrinsic noise). To analyse the behaviour of the system under external noise (i.e. a noisy input), we use a two-step signalling mechanism: a signalling molecule induces release of a second signalling molecule, which is subject to degradation and which acts as input and activates L1 (electronic supplementary material, equation (S6)). This set-up allows us to generate Poisson-distributed noise in the input, with mean increasing from 0 to 200 (as the first signalling molecule is increased from 0 to 200). The analysis of noise properties with and without extrinsic noise indicate that under the model parameters and extrinsic noise characteristics used here, the system is dominated by intrinsic noise (electronic supplementary material, figure S10). It should be noted, however, that this analysis considers only one specific type of external noise (Poisson). Scripts for both the stochastic models, executable with SPiM, are included in the electronic supplementary material.
